# Positive Murphy's Sign of Fitz-Hugh-Curtis Syndrome

**DOI:** 10.7759/cureus.78521

**Published:** 2025-02-04

**Authors:** Kotaro Kunitomo, Yukinori Harada, Takahiro Tsuji, Taro Shimizu

**Affiliations:** 1 General Medicine, National Hospital Organization (NHO) Kumamoto Medical Center, Kumamoto, JPN; 2 Diagnostic and Generalist Medicine, Dokkyo Medical University Hospital, Mibu, JPN

**Keywords:** availability bias, biliary disease, chlamydia trachomatis, diagnostic errors, fitz-hugh-curtis syndrome, murphy's sign, pelvic inflammatory disease

## Abstract

A Japanese woman in her 30s presented to the emergency department at midnight with right upper quadrant pain that had lasted for a week. Without a definitive diagnosis, she was prescribed acetaminophen and levofloxacin and discharged. When her pain persisted the next morning, she visited her primary care physician and reported fever and right upper quadrant pain. She was referred back to the emergency department with suspected cholecystitis.

The patient's vital signs were stable, including a temperature of 36.6°C. Physical examination revealed right upper abdominal tenderness and a positive Murphy's sign, but no other abdominal tenderness. Laboratory tests showed normal white blood cell count and liver enzymes and a slightly elevated C-reactive protein concentration (2.44 mg/dL). Abdominal ultrasound showed no abnormalities. Further questioning revealed a history of unprotected sex, lower abdominal pain before the right upper quadrant pain, and increased vaginal discharge. Urine polymerase chain reaction confirmed *Chlamydia trachomatis*. The patient was diagnosed with pelvic inflammatory disease, specifically Fitz-Hugh-Curtis syndrome (FHCS), and treated with ceftriaxone and minocycline. Her symptoms improved after seven days of treatment.

This case highlights the need for careful clinical evaluation and consideration of FHCS in patients presenting with right upper quadrant pain, especially when laboratory findings and imaging studies do not support biliary disease. A thorough history including symptoms of pelvic inflammatory disease, such as lower abdominal pain and vaginal discharge, is also necessary to accurately diagnose FHCS.

## Introduction

Murphy's sign is a clinical finding often associated with acute cholecystitis and is characterized by pain elicited during palpation of the right upper quadrant while the patient inhales deeply. While Murphy's sign has a high sensitivity (97%) for diagnosing acute cholecystitis, its low specificity (48%) necessitates careful interpretation, as a positive result does not definitively indicate acute cholecystitis [1].

Fitz-Hugh-Curtis syndrome (FHCS) is a rare complication of pelvic inflammatory disease (PID) characterized by perihepatic inflammation and adhesions between the liver capsule and the peritoneum. It has been reported to occur in 4-27% of women with PID [1]. It has been noted that the microorganisms associated with PID can spread in three ways: spontaneous ascending infection, hematogenous spread, and lymphatic spread [2]. It commonly presents with right upper quadrant pain and can mimic hepatobiliary and other abdominal diseases, as acute cholecystitis, cholelithiasis, right-sided pyelonephritis, pleurisy, and pulmonary embolism are the differential diagnoses [3-5]. Previous reports have documented cases of FHCS initially misdiagnosed as acute cholecystitis, leading to delays in appropriate treatment [6-8].

This case highlights the importance of comprehensive clinical evaluation, including a detailed history and consideration of atypical presentations, to distinguish FHCS from other causes of right upper quadrant pain. Early diagnosis is essential to prevent complications and achieve favorable outcomes in patients with FHCS.

## Case presentation

A Japanese woman in her 30s visited the emergency department with right upper quadrant abdominal pain. No definitive diagnosis was made at that time. She was prescribed acetaminophen and levofloxacin and discharged home. However, she visited her primary care physician because her symptoms did not improve by the next morning. She reported right upper quadrant abdominal pain and fever and was referred to the emergency department with a suspected diagnosis of cholecystitis. She had a history of endometriosis and no history of trauma. She had no nausea, vomiting, pain during urination, or frequent urination. Her last menstrual period was 14 days ago. Her temperature was 36.6°C, and other vital signs were normal. Physical examination revealed tenderness in the right upper abdomen and a positive Murphy's sign. No tenderness was noted in other areas of the abdomen. She also complained of pain during inhalation, but other aggravating and relieving factors were unknown. Her pregnancy test was negative. The blood test results are provided in Table 1.

**Table 1:** Laboratory test results and reference ranges

Parameter	Result (unit)	Reference range (unit)
White blood cell count	8.3×10³/μL	3.3-8.6×10³/μL
Aspartate aminotransferase	11 U/L	13-30 U/L
Alanine aminotransferase	9 U/L	7-23 U/L
Gamma-glutamyl transpeptidase	20 U/L	9-32 U/L
Alkaline phosphatase	60 U/L	38-113 U/L
C-reactive protein	2.44 mg/dL	<0.14 mg/dL

**Figure 1:**
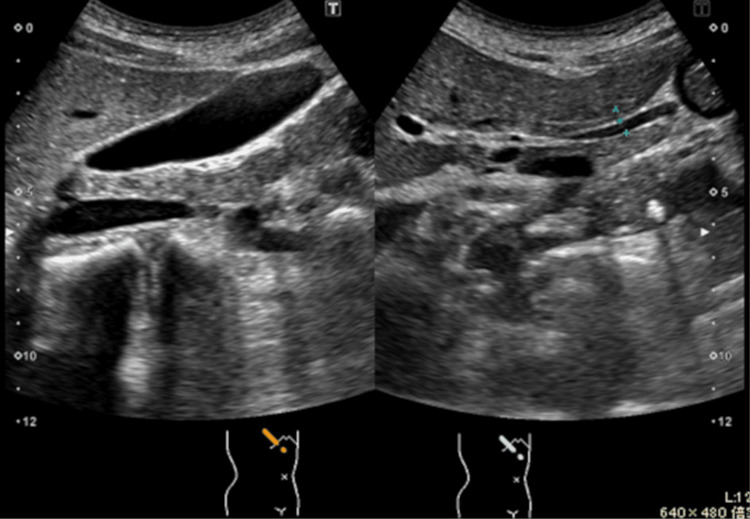
Abdominal ultrasound findings not suggestive of biliary disease

C-reactive protein was mildly elevated, but there were no other abnormalities. Abdominal ultrasonography showed no gallstones, gallbladder wall thickening, gallbladder enlargement, or bile duct dilatation (Figure 1).

At this time, we reevaluated the medical history of the patient. She reported unprotected sexual intercourse, lower abdominal pain that preceded the onset of pain in the right hypochondrium about a week earlier, and increased vaginal discharge. She noted that she had not reported the lower abdominal pain as it had resolved within a few days. Due to the increased likelihood of PID and FHCS, a polymerase chain reaction test of the urine for *Chlamydia trachomatis* was performed, yielding a positive result. Tests for syphilis, gonorrhea, and human immunodeficiency virus were negative. The patient was diagnosed with FHCS and treated with seven days of antimicrobial therapy (ceftriaxone 2 g/day and minocycline hydrochloride 200 mg/day). After completing treatment, her symptoms improved. We also recommended that her partner undergo testing and receive appropriate treatment.

## Discussion

In this case, the presence of a positive Murphy's sign initially led to the suspicion of biliary disease, especially acute cholecystitis. This illustrates the availability bias, a type of cognitive bias, where the physician, influenced by the prominent finding, linked Murphy's sign directly to acute cholecystitis. In addition, as the patient did not initially report lower abdominal pain, FHCS was not considered in the differential diagnosis.

FHCS is a rare complication of PID characterized by perihepatic adhesions between the liver capsule and the abdominal wall. These adhesions cause right upper quadrant pain, which is often exacerbated by respiration due to movement of the liver against the inflamed peritoneum [4]. This pain can mimic what is associated with biliary disease and result in a positive Murphy's sign, increasing the likelihood of misdiagnosis. Cases of FHCS with positive Murphy's sign have been reported, and FHCS can be misdiagnosed as acute cholecystitis in clinical practice [1,3]. In the absence of imaging findings suggestive of biliary disease despite a positive Murphy's sign, inflammation of the peritoneum on the surface of the liver should be considered. Recognizing the potential for FHCS to mimic biliary disease is essential to prevent delays in diagnosis and ensure timely, effective management. Early treatment of FHCS is important to minimize the risk of infertility [2].

Approximately half of patients with FHCS present with isolated right upper quadrant pain without lower abdominal pain [6,9], making the differentiation of biliary diseases challenging. However, lower abdominal pain, which is often associated with PID, is critical in diagnosing FHCS. Thus, despite transient symptoms suggestive of PID, such as lower abdominal pain and tenderness, obtaining a detailed patient history is essential [10]. In this case, the absence of findings indicative of biliary disease, such as abnormal imaging findings, created a discrepancy that prompted a reassessment of the initial diagnosis, ultimately leading to the diagnosis of FHCS.

While the differentiation between FHCS and cholecystitis can be difficult, imaging findings such as the presence of small, avascular peritoneal masses at the upper dome of the liver on ultrasound have been reported as a useful diagnostic feature for FHCS [11]. Although such findings were not observed in this case, the potential of non-invasive imaging modalities to aid in distinguishing FHCS from biliary disease is noteworthy.

## Conclusions

In a young woman with a positive Murphy's sign but no imaging findings suggestive of biliary disease, the diagnosis should be reconsidered to include inflammation of the peritoneum on the liver surface. Furthermore, for female patients with right upper quadrant abdominal pain, reevaluating the detailed history for signs of PID is crucial, as lower abdominal pain and tenderness may resolve during the clinical course. These approaches can help reduce diagnostic errors associated with FHCS.
